# Development of Low-Fat Soft Dough Biscuits Using Carbohydrate-Based Fat Replacers

**DOI:** 10.1155/2013/576153

**Published:** 2013-04-28

**Authors:** Bhawna Chugh, Gurmukh Singh, B. K. Kumbhar

**Affiliations:** ^1^Department of Food Science & Technology, G. B. Pant University of Agriculture and Technology, Uttarakhand, Pantnagar 263145, India; ^2^Department of Post Harvest Process and Food Engineering, G. B. Pant University of Agriculture and Technology, Uttarakhand, Pantnagar 263145, India

## Abstract

Experiments were conducted to develop low-fat soft dough biscuits using carbohydrate-based fat replacers (maltodextrin and guar gum). A central composite rotatable design was used to optimise the level of sugar 24–36%, composite fat (fat 10.5–24.5%, maltodextrin 10.4–24%, and guar gum 0.1–0.5%), ammonium bicarbonate 0.5–2.5%, and water 20–24% for production of low-fat biscuits. Diameter (*P* < 0.01) and stress-strain ratio (*P* < 0.05) decreased significantly with increase in the amount of sugar. There was a significant decrease (*P* < 0.01) in spread ratio at high amount of water. Hardness was significantly affected by the interactions of ammonium bicarbonate with sugar (*P* < 0.05) and fat (*P* < 0.1). The optimum level of ingredients obtained for low-fat biscuits was sugar 31.7 g, fat 13.55 g, maltodextrin 21.15 g, guar gum 0.3 g, ammonium bicarbonate 2.21 g, and water 21 mL based on 100 g flour. The fat level in the optimised low-fat biscuit formulation was found to be 8.48% as compared to 22.65% in control; therefore, the reduction in fat was 62.5%.

## 1. Introduction 

The Indian bakery industry is the largest of the food processing industries, estimated to be over $1,400 million. The major products within this industry include bread, cakes, pastries, and biscuits. Short dough biscuits are products made from soft and weak wheat flours and are characterised by a formula high in sugar and shortening. Fat in a biscuit formulation has a multifaceted function. It is the principal ingredient responsible for tenderness, keeping quality, grain, and texture, and adding richness to biscuits [1]. 

The food industry is primarily driven by consumer health trends. A present day dietary concern is the consumption of a large amount of fat and sugar. With the growing incidence of obesity and diabetes, low calorie foods have gained immense popularity. Most well-maintained strategies in terms of fat reduction diets involve either the use of low-fat foods or fat substitutes or modifications such as trimming of fat from foods. So, the use of fat mimetics instead of conventional fats and oils helps in reducing calorie intake, whereas fat substitutes are either resistant to digestive lipases or partially digested [2, 3]. Fat replacers are grouped broadly into either lipid-, carbohydrate-, or protein-based materials. Carbohydrate-based replacers incorporate water into a gel type structure, resulting in lubricant or flow properties similar to those of fats in some food systems. It is likely that desirable textures can be achieved using these types of substitutes, and there are few regulatory obstacles regarding any toxicological potential [2]. Maltodextrin can be used in a gram-for-gram fat replacement in bakery goods that provides 4 kcal or 16.8 kJ/g [4]. Maltodextrins generally have a DE (dextrose equivalent) between 3 and 20. The higher the DE value, the higher the solubility and sweetness. Gums are referred to as hydrophilic colloids which can fulfil some of the bulking properties of sucrose and fat. The replacement of 50% of fat by soluble *β*-glucan and amylodextrins derived from oat flour resulted in cookies not significantly different from the full-fat ones, but at higher substitution levels moistness and overall quality were decreased [5]. Also tenderness of biscuits decreased with the increase of fat substitution by pectin, gum, or oat-based fat mimetics [6]. The previous work on fat replacers indicated that polydextrose, maltodextrins, and Simplesse are the most appropriate as far as cookies properties are concerned, but the main problem noted is the high hardness of the biscuits [7]. Gallagher et al. [8] developed low-fat biscuits using sugar and fat replacers and reported their effect on biscuit dimensions, colour, and texture. Sudha et al. [9] also reported the effect of fat replacers, namely, maltodextrin and polydextrose, on the biscuit hardness.

Flour, sugar, fat, water, and salt are the main components in a biscuit formulation. Changes made to these principal components have significant effects on final biscuit quality [10, 11]. Fat level and type have a significant effect on the rheological characteristics of biscuit dough and on the properties of the baked biscuits [12]. Replacement of the sensory properties of fat is difficult in low moisture bakery foods like cookies with final moisture between 3 and 4% [13]. Sugar delivers sweetness, influences the structural and textural properties of cookies, and enhances incorporation of air into the fat during cookie dough preparation. Increasing water quantity produced a reduction of consistency and an increase in fluidity and in adhesiveness of dough. The quantity of water affected the behaviour of the dough after baking. A slight increase in biscuit length was observed when the water content was increased particularly from 21% upwards [14]. 

The main objective of this study was to develop low-fat biscuits using combinations of carbohydrate-based fat replacers. Moreover, to produce acceptable quality low-fat biscuit, the level of other ingredients, namely, sugar, ammonium bicarbonate, and water, was varied to take into account the synergetic effect on the physical and sensory parameters. The response surface methodology was used to minimise the number of baking trials while gathering all information relating to ingredient interactions and quality characteristics.

## 2. Materials and Methods

### 2.1. Materials

Refined wheat flour, whole wheat flour, sugar, sodium bicarbonate, ammonium bicarbonate, skim milk powder, vanilla essence, and hydrogenated fat were procured from local market. Liquid glucose was collected from Uttarakhand maize processing unit, SIDCUL, Rudrapur, Uttarakhand, India. Carbohydrate based fat replacers, maltodextrin (DE = 16) and guar gum, were procured from M/s Ensigns Healthcare Private Ltd., Pune, Maharashtra and Hindustan Gum and Chemicals Ltd., Bhiwani, Haryana, India.

### 2.2. Experimental Plan

Response surface methodology which involves design of experiments, selection of levels of variables in experimental runs, fitting mathematical models, and finally selecting variables' level by optimizing the response was employed in the study [15]. A central composite rotatable design (CCRD) was used to design the experiments comprising of four independent variables (Table 1). The parameters that influence the product quality and acceptability were taken as responses. The statistical software package Design-Expert 8.0.6 (Trial version), Stat-Ease Inc., Minneapolis, USA (http://www.statease.com/), was used to construct the experimental design as well as analyze the data. The experimental design and the codes for the processing variables have been reported in Table 1. A total of 32 combinations were generated for the four independent variables, and the experiments at centre point were repeated eight times. The magnitude of the effect of independent variables on the responses was based on magnitude of regression coefficients. 

### 2.3. Preparation of Biscuits

Biscuits were prepared using traditional creamery method given by Whitley [16]. For preparation of low-fat biscuits, sugar, fat, maltodextrin, guar gum, ammonium bicarbonate, and water were mixed in the quantities on 100 g flour (63.7 g white flour and 36.3 g whole wheat flour) basis as per the experimental design to form different formulations. In these formulations, fixed amounts of liquid glucose (3 g), skim milk powder (3 g), sodium bicarbonate (1.18 g), and vanilla essence (4 drops) were mixed. 

The control biscuit formulation contained the following ingredients at the indicated level: flour, 100 g (63.7 g white flour and 36.3 g whole wheat flour); liquid glucose, 3 g; skim milk powder, 3 g; sodium bicarbonate, 1.18 g; vanilla essence (4 drops); sugar, 26.5 g; fat, 35 g; ammonium bicarbonate, 1.22 g; water, 23 mL [16]. 

The dough was sheeted to 4 mm height biscuits which were baked in an air circulation oven at 190 ± 2°C for 8 min. The biscuits were cooled for 30 min at room temperature and stored in low density polyethylene packs before further analysis.

### 2.4. Physical and Chemical Evaluation of the Biscuits

The biscuit diameter and thickness were determined by AACC [17] methods. Spread ratio was then calculated as diameter divided by thickness of the biscuit. Crude fat was determined using standard AOAC [18] method.

### 2.5. Texture Analysis

Hardness of biscuits was measured by Stable Micro Systems Texture Analyzer (TAXT 2i). It was measured in terms of maximum force used to break the biscuit sample. The biscuits were placed under sharp blade cutting probe, 70 mm long and 0.4 mm thick. The analyzer was set at a “return to start” cycle, a speed of 1 mm/s and a distance of 3 mm, and pretest speed 5 mm/s and posttest speed 10 mm/s. The maximum force was expressed in N. Stress was calculated by dividing the maximum force by area of blade, and strain was expressed as the maximum distance travelled by probe to break the biscuit. All measurements were replicated six times, and the mean values are reported.

### 2.6. Sensory Evaluation

Ten semitrained panelists carried sensory evaluation of low-fat soft dough biscuits and compared with the control samples. Three 1-hour preliminary sessions were conducted to train the panelists so as to familiarize themselves with the samples. In the first session, the subjects described two very different biscuits (control and low-fat biscuit) and mainly focussed on the texture change. In the second session, the most frequently cited attributes were selected, and their definitions and the protocols scoring them were developed. During the third session, the panel lists were able to understand the test and were given a score sheet to evaluate sensory attributes, namely, colour, taste, texture, flavor, and overall acceptability (OAA), and asked to score samples on 5-point scale where scores 1, 2, 3, 4, and 5 represented poor, fair, satisfactory, good, and excellent, respectively [19]. Panelists were instructed to cleanse their palate with tap water before tasting each sample. Product characterization was carried out under “day light” illumination and in isolated booths within a sensory laboratory. 

### 2.7. Data Analysis

The experimental data were analysed using second-order model given below:

(1) $$\begin{array}{c} y=\beta_{0}+\sum_{i=1}^{4}\beta_{i}x_{i}+\sum_{i=1}^{4}\beta_{ii}x_{i}^{2}+\sum_{i=1}^{3}\sum_{j=i+1}^{4}\beta_{ij}x_{i}x_{j}, \end{array}$$

where *y* = response, *x*_*i*_, *x*_*j*_ = coded processing parameters, and *β*_0_, *β*_*i*_, *β*_*ii*_, *β*_*ij*_ = regression coefficients.

Adequacy of the model was determined using coefficient of determination (*R* ^2^), *F*-value, and adequacy of precision. The effect of variables at linear, quadratic and interactive levels on the response was described using various levels of significance.

Numerical optimization technique of the Design-Expert (8.0.6) software was used for simultaneous optimization of the multiple responses, and for this some constraints had to be decided. These constraints set the guidelines to get the desired results. The goal seeking begins at a random starting point and proceeds up or down the steepest stop on the response surface for a maximum or minimum value of a response, respectively. The response values and the analysis of the models gave the valuable information in deciding constraints for independent variables and responses. Therefore, all the independent variables were kept within experimental range except composite fat which was kept at minimum as our main goal is to produce low-fat biscuit. The multiple responses, namely, spread ratio, hardness, stress-strain ratio, and overall acceptability (OAA), were considered for optimization as they represent quality attributes adequately. The numerical optimization finds a point that maximizes the desirability function.

## 3. Results and Discussion

The experimental results for physical and sensory parameters are reported in Table 1. The product with varied formulations had diameter, thickness, spread ratio, hardness, and stress-strain ratio in the ranges of 6.08–7.03 cm, 0.57–0.73 cm, 8.44–11.96, 25.07–84.02 N, and 0.574–3.19, respectively. The sensory score ranged 3.00–4.83, 3.25–4.25, 2.88–4.25, 2.88–4.13, and 2.36–4.25 for colour, texture, taste, flavor, and OAA, respectively. The corresponding values of physical parameters for control biscuits were 5.98 cm, 0.62 cm, 9.64, 32.52 N, and 0.73, respectively, while for sensory parameters were 4.5, 4.0, 3.33, 3.83, and 4.02, respectively. Most of the combinations were better than the control formulation.

The Design-Expert software was used to fit the second-order response surface model (1) into the experimental data of all responses using regression analysis, and the resulting predictive equations are given below. All models have adequate precision ratio of more than 4, thus indicative of the fact that the experiments were carried out with adequate precision, and moreover, the *F*-value was found significant in all models:

(2) diameter  (y)=6.5537+0.1391∗X1Diameter  (y) +0.0925∗X2+0.1575∗X3Diameter  (y) −0.0025∗X4−0.0137∗X1∗X2Diameter  (y) +0.0725∗X1∗X3+0.0087∗X1∗X4Diameter  (y) −0.058∗X2∗X3+0.0475∗X2∗X4Diameter  (y) +0.0462∗X3∗X4+0.0015∗X1∗X1Diameter  (y) −0.0146∗X2∗X2−0.0009∗X3∗X3Diameter  (y) +0.0353∗X4∗X4 (R2=79.84%),thickness  (y)=0.605+0.0120∗X1Thickness(y) −0.0087∗X2−0.0054∗X3Thickness(y) +0.0229∗X4+0.0056∗X1∗X2Thickness(y) −0.0243∗X1∗X3+0.0081∗X1∗X4Thickness(y) −0.0043∗X2∗X3−0.0143∗X2∗X4Thickness(y) +0.0131∗X3∗X4+0.0169∗X1∗X1Thickness(y) +0.0182∗X2∗X2−0.0017∗X3∗X3Thickness(y) +0.0182∗X4∗X4 (R2=79.72%),spread  ratio(y) =10.8368+0.0424∗X1  +0.2621∗X2+0.3467∗X3  −0.366∗X4−0.1224∗X1∗X2  +0.4960∗X1∗X3−0.1276∗X1∗X4  −0.0487∗X2∗X3+0.2583∗X2∗X4  −0.1369∗X3∗X4−0.2708∗X1∗X1  −0.2998∗X2∗X2+0.0376∗X3∗X3  −0.2269∗X4∗X4 (R2=75.66%),hardness  (y)=68.6025−4.2627∗X1Hardness  (y) +3.2931∗X2−0.7124∗X3Hardness  (y) +1.3522∗X4−4.075∗X1∗X2Hardness  (y) −6.1027∗X1∗X3+2.1093∗X1∗X4Hardness  (y) +5.2673∗X2∗X3+1.037∗X2∗X4Hardness  (y) −1.5337∗X3∗X4−7.5804∗X1∗X1Hardness  (y) −3.4916∗X2∗X2−5.9171∗X3∗X3Hardness  (y) −7.3353∗X4∗X4 (R2=77.71%),stress-strain  ratio  (y) =1.6139−0.2123∗X1  +0.1394∗X2+0.0056∗X3  +0.0559∗X4−0.1659∗X1∗X2  −0.2748∗X1∗X3+0.1132∗X1∗X4  +0.2355∗X2∗X3+0.0017∗X2∗X4  −0.0656∗X3∗X4−0.1336∗X1∗X1  −0.0132∗X2∗X2−0.1012∗X3∗X3  −0.1424∗X4∗X4 (R2=62.87%),colour(y)=3.5537+0.0529∗X1Colour(y) +0.0354∗X2−0.2437∗X3Colour(y) +0.0754∗X4+0.1718∗X1∗X2Colour(y) −0.0356∗X1∗X3+0.0631∗X1∗X4Colour(y) −0.1256∗X2∗X3+0.1281∗X2∗X4Colour(y) −0.1343∗X3∗X4+0.0718∗X1∗X1Colour(y) +0.0443∗X2∗X2−0.0306∗X3∗X3Colour(y) −0.0306∗X4∗X4 (R2=72.0%),texture(y)=3.5437−0.0637∗X1Texture(y) −0.1162∗X2−0.142∗X3Texture(y) −0.072∗X4+0.0143∗X1∗X2  +0.0081∗X1∗X3−0.0168∗X1∗X4  +0.0218∗X2∗X3−0.0031∗X2∗X4  +0.0156∗X3∗X4+0.0887∗X1∗X1  −0.0137∗X2∗X2+0.0187∗X3∗X3  +0.0462∗X4∗X4 (R2=71.0%),taste(y)=3.4575−0.08417∗X1Taste(y) −0.1358∗X2−0.1066∗X3Taste(y) −0.0525∗X4−0.0087∗X1∗X2Taste(y) +0.1562∗X1∗X3−0.0975∗X1∗X4Taste(y) +0.1787∗X2∗X3−0.0125∗X2∗X4Taste(y) −0.0275∗X3∗X4+0.05∗X1∗X1Taste(y) −0.0125∗X2∗X2−0.0462∗X3∗X3Taste(y) −0.0012∗X4∗X4 (R2=79.27%),flavour(y)=33.3825−0.0629∗X1Flavour(y) −0.1212∗X2−0.1437∗X3Flavour(y) −0.012∗X4−0.0868∗X1∗X2Flavour(y) +0.1218∗X1∗X3−0.0743∗X1∗X4Flavour(y) +0.1043∗X2∗X3−0.0068∗X2∗X4Flavour(y) −0.0356∗X3∗X4+0.0742∗X1∗X1Flavour(y) −0.0132∗X2∗X2−0.0332∗X3∗X3Flavour(y) +0.0205∗X4∗X4 (R2=72.97%),overall  acceptability  (y) =3.4525−0.0925∗X1  −0.1925∗X2−0.225∗X3  +0.0216∗X4−0.0212∗X1∗X2  +0.1125∗X1∗X3−0.0625∗X1∗X4  +0.1125∗X2∗X3−0.0225∗X2∗X4  −0.0412∗X3∗X4+0.0495∗X1∗X1  +0.002∗X2∗X2−0.1354∗X3∗X3  +0.042∗X4∗X4 (R2=82.65%).

Based on the regression analysis, the results are discussed below.

### 3.1. Effect of Independent Variables on Different Responses

#### 3.1.1. Effect of Sugar

The level of sugar had a significant effect on all the responses except spread ratio. The effect of sugar was significant on diameter (*P* < 0.01), thickness (*P* < 0.1), hardness (*P* < 0.1), and stress-strain ratio (*P* < 0.05) at linear level. It affected thickness (*P* < 0.01), spread ratio (*P* < 0.05), and hardness (*P* < 0.01) at quadratic level also. Diameter and thickness increased with increase in sugar level, while hardness and stress-strain ratio decreased. Similar findings were also reported by Pareyt et al. [20] who found increase in spread of biscuits with increase in the level of sugar. Higher sucrose levels in the cookie dough recipe lead to increased sucrose dissolution during baking. This results in higher quantities of solvent phase, and as a consequence, spread increases. 

It was observed that sugar had a significant effect on texture (*P* < 0.1), taste (*P* < 0.05), and OAA (*P* < 0.05) scores. Moreover, the sugar also affected significantly texture (*P* < 0.05) and flavour (*P* < 0.1) quadratically.

The overall effect of sugar was found significant on all physical characteristics as it affected taste, flavor, and OAA significantly at *P* < 0.01, *P* < 0.05, and *P* < 0.1, respectively.

#### 3.1.2. Effect of Composite Fat (Fat and Fat Replacers)

It was found that combination of fat and fat replacers significantly affected the diameter (*P* < 0.01) and spread ratio (*P* < 0.05). Diameter and spread ratio increased with increase in the level of fat replacer (maltodextrin and guar gum). Sudha et al. [9] also reported that replacement of fat with maltodextrin at different levels had improving effect on the spread and texture of the biscuits. The quadratic term of fat was significant for thickness (*P* < 0.01), spread ratio (*P* < 0.05), and hardness (*P* < 0.1) while insignificant for other responses. There was a significant (*P* < 0.01) effect of composite fat on all the sensory responses except colour.

The overall effect of composite fat was significant on all physical responses of biscuit, namely, diameter, thickness, spread rati,o and hardness, except stress-strain ratio. The overall effect of composite fat was significant on all the sensory parameters except texture. The overall effect of fat was observed to be more pronounced on OAA (*P* < 0.01) and taste (*P* < 0.01) than on flavour (*P* < 0.05) followed by colour (*P* < 0.1). 

#### 3.1.3. Effect of Ammonium Bicarbonate

Ammonium bicarbonate had significant effect on diameter (*P* < 0.01) and spread ratio (*P* < 0.05). With increase in the level of ammonium bicarbonate, diameter and spread ratio of product increased. Similar findings were reported by Finney et al. [21]. However, the quadratic term of ammonium bicarbonate had significant effect on hardness (*P* < 0.1). 

 The total effect of ammonium bicarbonate on all the sensory parameters was significant at *P* < 0.01 except taste (*P* < 0.05). The ammonium bicarbonate affected the OAA significantly (*P* < 0.01) at quadratic level. 

The overall effect of ammonium bicarbonate was found significant on all the physical and textural parameters, and it was maximum on diameter (*P* < 0.01). The overall effect of ammonium bicarbonate on all sensory parameters was found to be significant at *P* < 0.01, except on texture, where the level of significance was *P* < 0.05.

#### 3.1.4. Effect of Water

Water had a significant (*P* < 0.01) effect on thickness and spread ratio both at linear and quadratic levels. Maache-Rezzoug et al. [14] reported the reduction in thickness and weight of biscuits with increase in the water concentration. It also affected thickness (*P* < 0.01), hardness (*P* < 0.01), spread ratio (*P* < 0.1), and stress-strain ratio (*P* < 0.1) quadratically.

Amongst the sensory parameters, only texture was significantly (*P* < 0.1) affected by water. It decreased with increase in water level. The overall effect of water on thickness was significant at *P* < 0.01, while it was significant at *P* < 0.05 on spread ratio and hardness. It was found that the overall effect of water on all the sensory responses was insignificant.

#### 3.1.5. Synergistic Effect of Independent Variables

**Figure 1:**
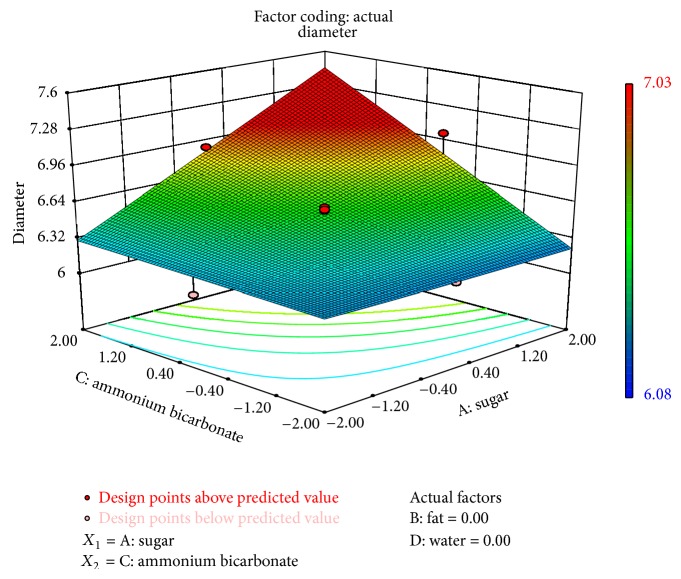
Surface plot representing the effect of sugar (*X*_1_) and ammonium bicarbonate (*X*_3_) on diameter of the biscuits.

**Figure 2:**
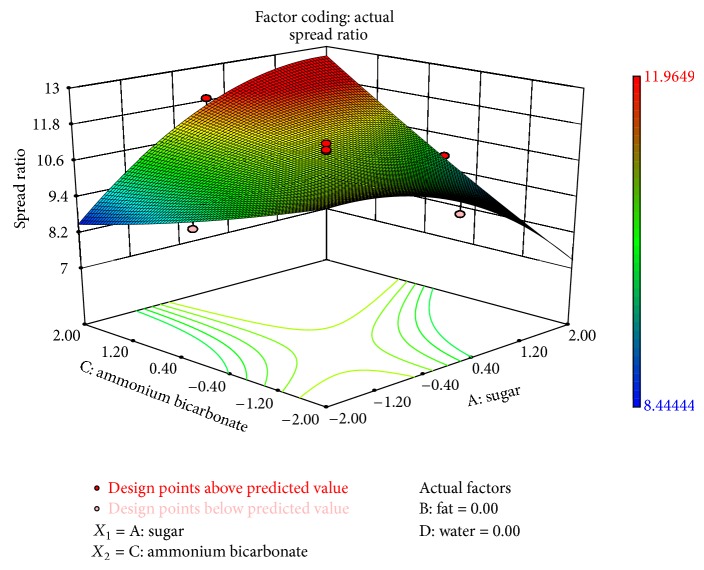
Surface plot representing the effect of sugar (*X*_1_) and ammonium bicarbonate (*X*_3_) on spread ratio of the biscuits.

**Figure 3:**
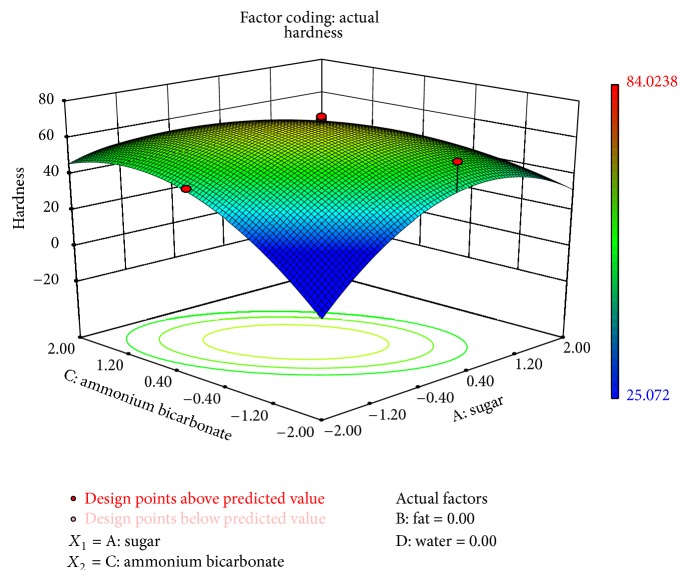
Surface plot representing the effect of sugar (*X*_1_) and ammonium bicarbonate (*X*_3_) on hardness of the biscuits.

The interaction of sugar and ammonium bicarbonate affected all physical characteristics significantly, while interaction of composite fat and water had a significant effect on thickness (*P* < 0.1) and spread ratio (*P* < 0.1). The interaction between ammonium bicarbonate and water was significant on thickness. Figures 1–3 showed the effect of sugar and ammonium bicarbonate on diameter, spread ratio, and hardness, respectively. Diameter and spread ratio increased with increase in sugar and ammonium bicarbonate levels as shown in Figures 1 and 2, respectively. A similar finding was also reported by Finney et al. [21]. Maache-Rezzoug et al. [14] also showed the positive correlation between sugar content and length. Hardness was maximum around the centre level of both sugar and ammonium bicarbonate (Figure 3). Stress-strain ratio was found to be significantly (*P* < 0.05) affected by the interaction of ammonium bicarbonate with sugar. The interaction of sugar and ammonium bicarbonate was found to be more significant for taste (*P* < 0.01) than flavour and OAA (*P* < 0.05). The interactive effect of water and sugar was found significant on taste (*P* < 0.05).

**Figure 4:**
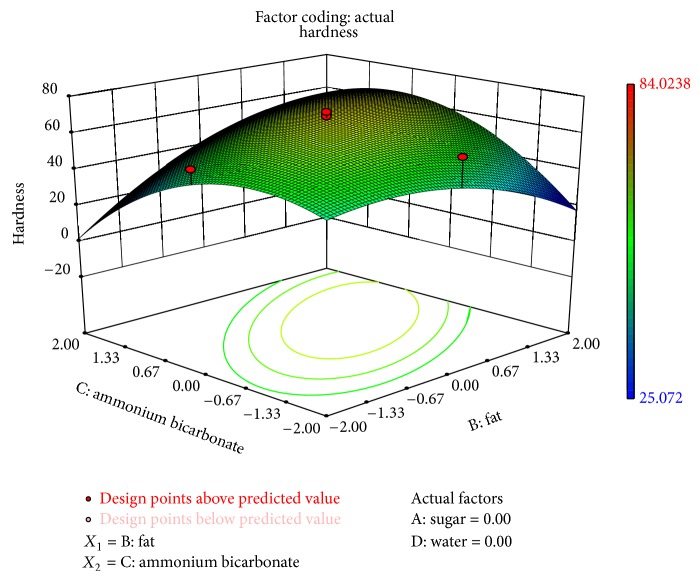
Surface plot representing the effect of fat (*X*_2_) and ammonium bicarbonate (*X*_3_) on hardness of the biscuits.

**Figure 5:**
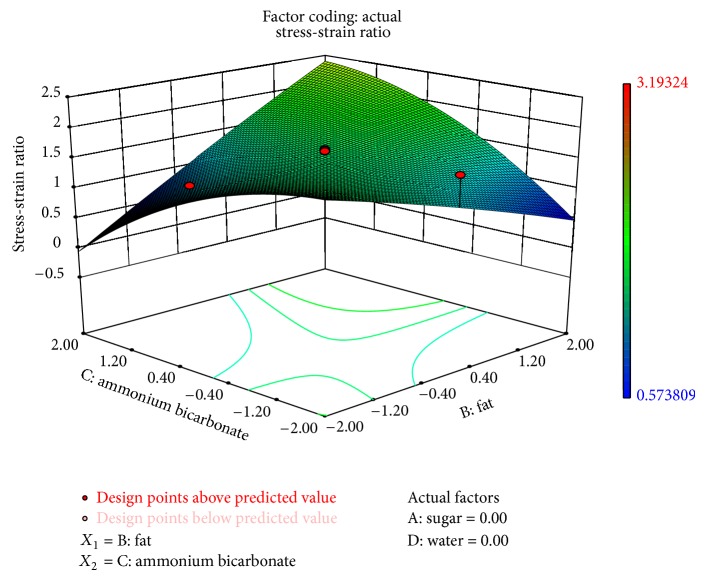
Surface plots representing the effect of fat (*X*_2_) and ammonium bicarbonate (*X*_3_) on stress-strain ratio of the biscuits.

Hardness (*P* < 0.1) and stress-strain ratio (*P* < 0.05) were significantly affected by ammonium bicarbonate and fat as shown in Figures 4 and 5.  Figure 5 shows that the hardness increased as the level of fat in the formulation decreased. It can also be concluded that the hardness increased at higher values of fat replacer (maltodextrin and guar gum). Sudha et al. [9] also demonstrated the effect of maltodextrin on the breaking strength of biscuit. They reported that force required to break biscuits containing 70% less fat was almost three times more than that required to break the control biscuits. Mamat et al. [22] also reported higher hardness for a biscuit with lower-fat content than a biscuit with normal-fat content. The stress-strain ratio (*σ*_max_/*ɛ*_max_) is related to brittleness of the sample [23, 24]. Zoulias et al. [25] also reported the increase in stress-strain ratio of cookies by replacement of fat with fat mimetics which resulted in the production of brittle cookies. Cookies that present a high ratio of *σ*_max_/*ɛ*_max_ are less compressible, more brittle and break easily. Brittleness can be considered a pleasant sensorial characteristic for the cookies as far as it does not become extremely great. 

**Figure 6:**
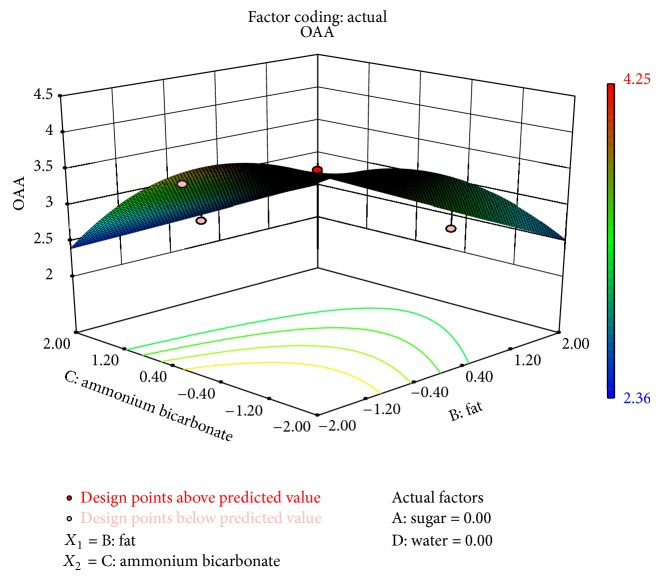
Surface plot representing the effect of fat (*X*_2_) and ammonium bicarbonate (*X*_3_) on overall acceptability of the biscuits.

The level of ammonium bicarbonate and fat also had a significant effect on OAA (Figure 6). There was a decrease in OAA at higher levels of fat replacers. A similar effect of fat replacers on cookies was reported by Zoulias et al. [7]. They found that the low-fat cookies had significantly lower flavour and general acceptance scores than the control cookies. It was observed that the effect of interaction of ammonium bicarbonate and composite fat was significant on colour, taste, and flavour. 

**Figure 7:**
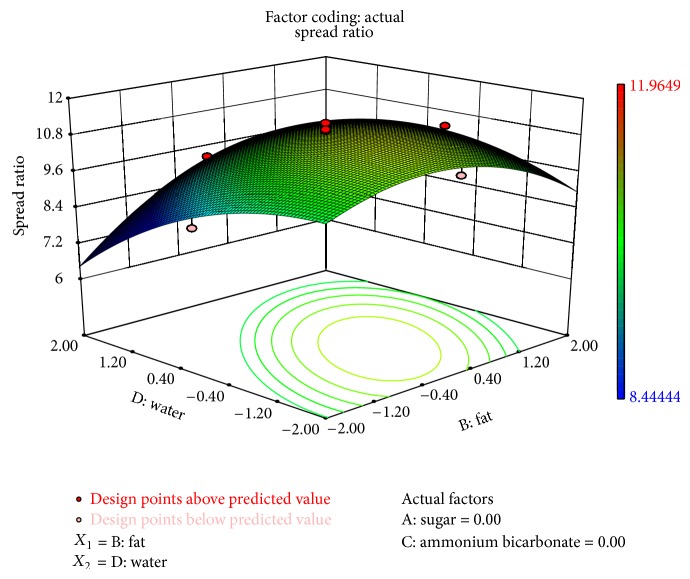
Surface plot representing the effect of fat (*X*_2_) and water (*X*_4_) on spread ratio of the biscuits.

The effect of interaction of fat and water was significant on thickness (*P* < 0.1) and spread ratio (*P* < 0.1). Spread ratio decreased with increase in water and fat replacer level (Figure 7). It was noticed that colour score decreased significantly (*P* < 0.1) by increase in the levels of fat replacers and water. Low-fat biscuits were found to be darker than the control biscuits because of the higher degree of the Maillard browning reactions which might be the result of carbohydrate nature of these fat replacers. A similar finding was also reported by Sanchez et al. [26] who found high colour intensity in biscuits made from carbohydrate-based fat replacers. It was also observed that the interaction of sugar and fat significantly affected colour (*P* < 0.05) and flavour (*P* < 0.1).

The interaction between ammonium bicarbonate and water significantly affected thickness. Goldstein and Seetharaman [27] correlated the increase in cookie height with increasing moisture content in the samples. Also, colour was found to be significantly (*P* < 0.1) affected by the interaction of ammonium bicarbonate with water.

#### 3.1.6. Combined Effect of Independent Variables

ANOVA (Tables 2 and 3) is used to show the total effect of variables individually and combination of all variables at linear, quadratic and interactive levels. It was found that all independent variables had significant effect on diameter (*P* < 0.01), thickness (*P* < 0.01), and spread ratio (*P* < 0.01) at linear level. They affected thickness and spread ratio quadratically at *P* < 0.01 and *P* < 0.05, respectively. At interactive level, they affected spread ratio (*P* < 0.1), hardness (*P* < 0.1), stress-strain ratio (*P* < 0.1), and thickness (*P* < 0.05).

The combined effect of independent variables was found on all sensory parameters at linear, quadratic, and interactive levels. They affected all parameters linearly at *P* < 0.01. At quadratic level, they affected texture (*P* < 0.1) and OAA (*P* < 0.05). At interactive level, they significantly affected colour (*P* < 0.05), flavour (*P* < 0.05), and taste (*P* < 0.01).

### 3.2. Optimization of Independent Variables for Low-Fat Biscuits

Design-Expert (8.0.6 trial version) software was employed to optimise ingredient level based on maximum spread ratio and OAA and minimum stress-strain ratio and composite fat (fat, maltodextrin, and guar gum) of biscuits using numerical methods of optimization. The optimum condition for different parameters obtained was sugar of 31.74 g, fat of 13.55 g, maltodextrin of 21.15 g, guar gum of 0.3 g, ammonium bicarbonate of 2.21 g, and water of 21 mL. The levels were based on 100 g flour. The comparison between predicted and experimental results is shown in Table 4. It shows a good agreement between predicted and experimental values. 

## 4. Conclusions

RSM was successfully used for optimizing different ingredients for the manufacture of low-fat soft dough biscuits. Results of the study indicate that hardness increases with increase in level of sugar and fat replacers and decrease in fat level. With increase in ammonium bicarbonate, diameter and spread ratio increase. Interactive effect of increased fat and water level decreases spread ratio. The optimized product had 62.5% replacement of fat with carbohydrate-based fat replacers, maltodextrin and guar gum.

## Figures and Tables

**Table 1:** Experimental design matrix for manufacture of low-fat biscuits.

Expt. No.	Coded form				Physical parameters					Sensory parameters				
	*X*_1_	*X*_2_	*X*_3_	*X*_4_	Diameter (cm)	Thickness (cm)	Spread ratio	Hardness (N)	Stress-strain ratio	Colour	Texture	Taste	Flavour	Overall acceptability
1	−1	−1	−1	−1	6.28	0.64	9.81	49.32	1.660	3.72	3.89	**4.25**	4.03	4.14
2	+1	−1	−1	−1	6.42	0.64	10.03	36.59	0.760	3.50	4.08	3.75	3.83	3.79
3	−1	+1	−1	−1	6.65	*0.57 *	11.67	*25.07 *	*0.574 *	3.20	4.00	3.40	3.60	3.30
4	+1	+1	−1	−1	6.55	**0.73**	8.97	39.82	1.067	4.25	3.75	3.25	3.25	3.38
5	−1	−1	+1	−1	6.63	0.63	10.52	35.53	0.829	3.75	4.00	3.13	3.13	3.13
6	+1	−1	+1	−1	6.82	*0.57 *	**11.96**	31.62	0.796	3.40	3.50	3.40	3.40	3.40
7	−1	+1	+1	−1	6.42	0.67	9.58	**84.02**	**3.193**	3.20	3.30	3.47	3.36	3.31
8	+1	+1	+1	−1	6.88	0.59	11.66	31.85	0.669	3.17	3.40	3.70	3.40	3.16
9	−1	−1	−1	+1	6.28	0.62	10.13	27.92	0.578	4.00	**4.25**	4.13	**4.13**	**4.25**
10	+1	−1	−1	+1	*6.08 *	0.72	*8.44 *	50.66	1.508	3.75	3.95	3.60	3.70	3.97
11	−1	+1	−1	+1	6.36	0.65	9.78	53.30	1.417	4.17	3.75	3.92	4.00	4.04
12	+1	+1	−1	+1	6.70	0.67	10.00	40.16	1.035	**4.83**	3.45	2.92	3.17	3.04
13	−1	−1	+1	+1	6.28	0.72	8.72	42.92	1.312	3.10	3.60	3.35	3.20	3.45
14	+1	−1	+1	+1	7.02	0.72	9.75	32.23	0.586	3.50	3.50	3.25	3.42	3.33
15	−1	+1	+1	+1	6.88	0.64	10.75	61.90	1.995	3.10	3.53	3.33	3.39	3.00
16	+1	+1	+1	+1	6.83	0.67	10.19	42.67	1.022	3.75	3.50	3.25	3.00	3.25
17	−*α*	0	0	0	6.12	0.65	9.42	48.08	1.396	3.92	3.92	3.58	3.50	3.71
18	+*α*	0	0	0	**7.03**	0.71	9.90	34.12	0.906	3.60	3.75	3.50	3.58	3.25
19	0	−*α*	0	0	6.32	0.72	8.78	55.70	1.531	4.10	3.60	3.70	3.50	3.70
20	0	+*α*	0	0	6.70	0.65	10.31	59.22	1.734	3.20	*3.25 *	*2.88 *	*2.88 *	2.88
21	0	0	−*α*	0	6.23	0.63	9.89	62.01	1.698	3.70	3.71	3.21	3.12	3.12
22	0	0	+*α*	0	6.90	0.58	11.90	33.50	0.864	*3.00 *	3.40	3.10	3.10	*2.36 *
23	0	0	0	−*α*	6.67	0.64	10.42	38.38	0.756	3.40	4.00	3.50	3.40	3.50
24	0	0	0	+*α*	6.75	**0.73**	9.25	45.64	1.475	3.30	3.33	3.17	3.25	3.40
25	0	0	0	0	6.50	0.58	11.21	68.04	1.587	3.50	3.60	3.50	3.50	3.50
26	0	0	0	0	6.57	0.60	10.95	70.11	1.647	3.60	3.50	3.40	3.40	3.40
27	0	0	0	0	6.50	0.60	10.83	67.04	1.555	3.60	3.50	3.55	3.30	3.45
28	0	0	0	0	6.60	0.62	10.65	67.77	1.582	3.50	3.55	3.50	3.40	3.50
29	0	0	0	0	6.50	0.60	10.83	69.14	1.657	3.50	3.60	3.40	3.40	3.44
30	0	0	0	0	6.60	0.60	11.00	71.54	1.648	3.60	3.53	3.35	3.35	3.40
31	0	0	0	0	6.58	0.62	10.61	67.98	1.624	3.55	3.50	3.56	3.31	3.50
32	0	0	0	0	6.58	0.62	10.61	67.20	1.611	3.58	3.57	3.40	3.40	3.43

*X*_1_: sugar, *X*_2_: composite fat, *X*_3_: ammonium bicarbonate, *X*_4_: water, MD: maltodextrin, GG: guar gum, A.B.: ammonium bicarbonate; figures in bold are maximum values, and figures in italics are minimum values.

**Table 2:** ANOVA for the overall effect of processing parameters on the physical and textural responses^a^.

Responses	Mean squares				
	Diameter (cm)	Thickness (cm)	Spread ratio	Hardness (N)	Stress-strain ratio
Total individual effect of processing parameters					

Sugar	0.1106∗∗∗	0.0046∗∗∗	1.3295∗∗	613.2477∗∗∗	0.6928∗∗
Fat	0.0612∗	0.0031∗∗	1.1300∗∗	269.4439∗	0.3601
Ammonium bicarbonate	0.1537∗∗∗	0.0026∗∗	1.4404∗∗	424.7674∗∗	0.4937∗
Water	0.0217	0.0059∗∗∗	1.2738∗∗	353.4363∗∗	0.1897

Combined effect of all processing parameters					

Linear level	0.3164∗∗∗	0.0046∗∗∗	1.9498∗∗∗	188.1162	0.4062
Quadratic level	0.0108	0.0070∗∗∗	1.5964∗∗	171.7865	0.3586
Interactive level	0.0356	0.0029∗∗	0.9737∗	238.5898∗	0.4685∗

^a^Significant at ∗10%, ∗∗5%, and ∗∗∗1%.

**Table 3:** ANOVA for the overall effect of processing parameters on the sensory responses^a^.

Responses	Mean squares				
	Colour	Texture	Taste	Flavour	Overall acceptability
Total individual effect of processing parameters					

Sugar	0.1553	0.0678	0.1575∗∗∗	0.1409∗∗	0.1100∗
Fat	0.2152∗	0.0681	0.1924∗∗∗	0.1307∗∗	0.2214∗∗∗
Ammonium bicarbonate	0.4030∗∗∗	0.1015∗∗	0.25∗∗∗	0.1921∗∗∗	0.4377∗∗∗
Water	0.1559	0.0393	0.0465	0.0251	0.0322

Combined effect of all processing parameters					

Linear level	0.4149∗∗∗	0.2577∗∗∗	0.238∗∗∗	0.2368∗∗∗	0.5802∗∗∗
Quadratic level	0.0665	0.0779∗	0.0354	0.0532	0.1666∗∗
Interactive level	0.2267∗∗	0.0034	0.1782∗∗∗	0.107∗∗	0.0850

^a^Significant at ∗10%, ∗∗5%, and ∗∗∗1%.

**Table 4:** Verification of the models by comparing the experimental values with the predicted values.

Response	Predicted value	Experimental value∗
Spread ratio	11.92	10.93 ± 0.06
Hardness, N	25.07	31.47 ± 1.02
Stress-strain ratio	0.409	0.599 ± 0.017
Overall acceptability	4.53	4.17 ± 0.41

∗Average of ten experiments.
